# Supplementary material for: Identification and initial characterization of novel prion protein gene (PRNP) variants in wild Montana mule deer
Source: Front Vet Sci. 2026 Jul 14;13:1861004. doi: 10.3389/fvets.2026.1861004 (PMC13407091; doi:10.3389/fvets.2026.1861004)
Supplement: Supplementary file 1 [file Data_Sheet_1.PDF]

Supplementary Materials for:

Novel Prion Protein Gene (*PRNP)* Variants in Wild Montana Mule Deer

Alyssa L. Seerley[1], Michael T. Rothfuss[2], Bridget M. Gray[1], Mylene A. Sebogo[1], Bethelhem A. Manakelew[1], June I. Pounder[1], Bruce E. Bowler[2], Moses J. Leavens[1], Andrea L. Grindeland Panter[1]

[1]Weissman Hood Institute at Touro University; McLaughlin Research Institute for Biomedical Sciences, Great Falls, MT
[2]University of Montana, Department of Chemistry & Biochemistry, Missoula, MT

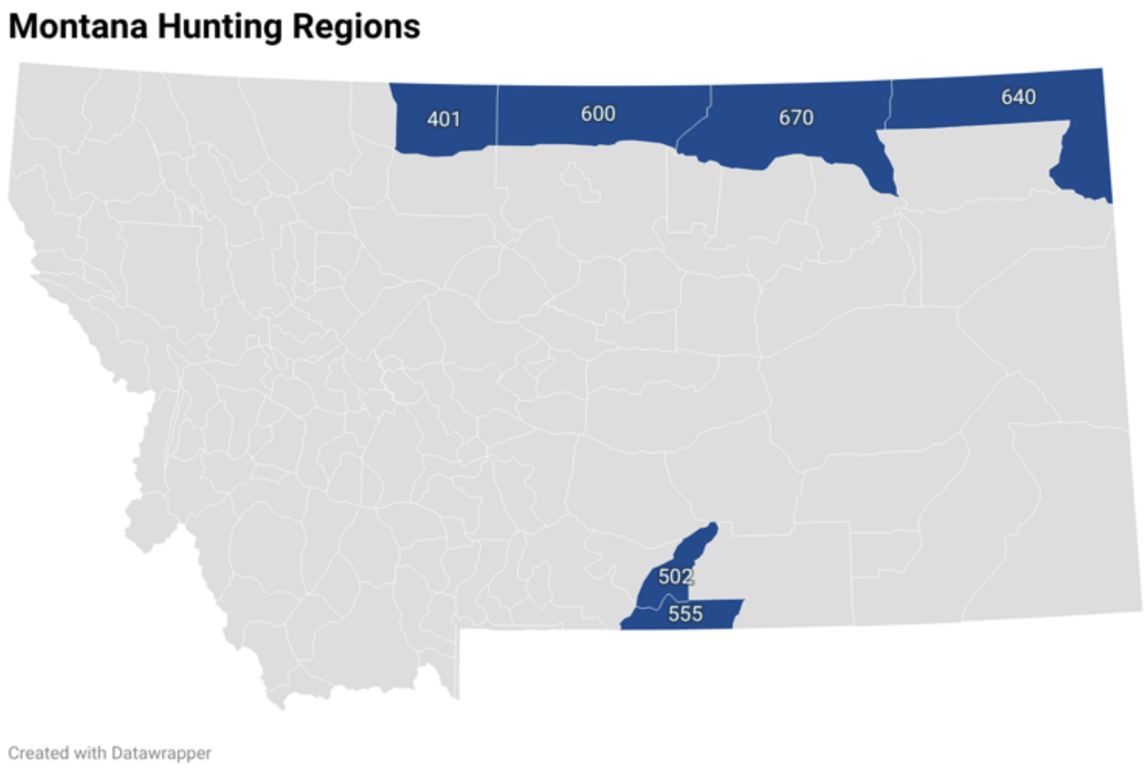

Figure S1. Montana hunting regions from which cervid samples in this dataset were

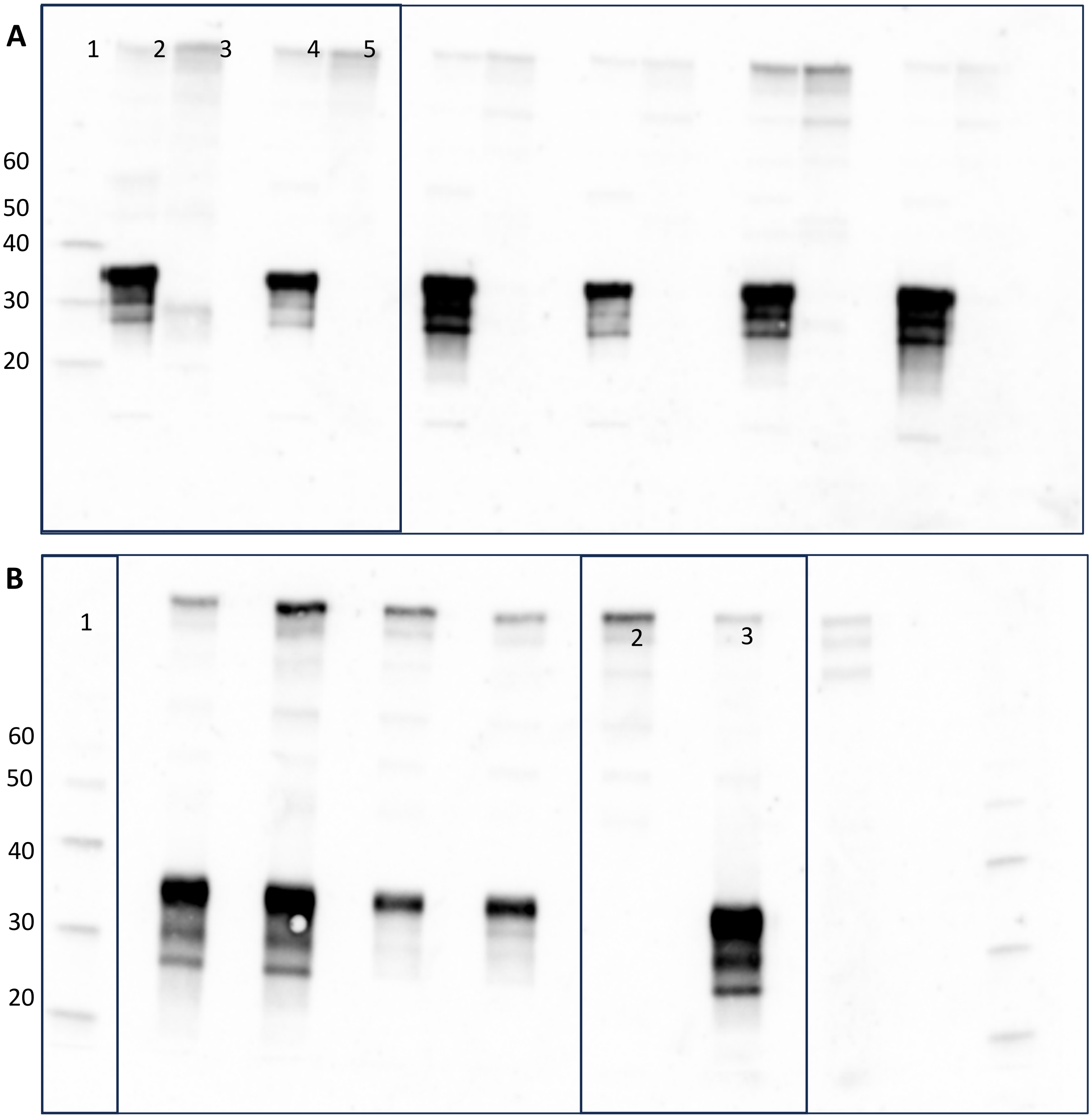

Figure S2: Transgenic mouse validation of PrP expression and infection using Western blot probed with 6D11. **[A]** Lane 1 is MagicMark XP standard. Lane 2 is CWD inoculated Cer-PrP-225SS (Tg1536+/+). Lane 3 is CWD Cer-PrP-225SS (Tg1536+/+) with PK treatment to display PK resistant prions. Lane 4 is normal brain homogenate (NBH) inoculated Cer-PrP-225SS (Tg1536+/+) control. Lane 5 is NBH Cer-PrP-225SS (Tg1536+/+) with PK treatment displaying the absence of PK resistant prions. **[B]** Lane 1 is MagicMark XP standard, lane 2 is PrP KO and lane 3 is FVB/NJ expressing endogenous mouse PrP only (no cervid PrP).

5’-
CATATGAAAAAGAGGCCAAAGCCGGGTGGTTGGAACACCGGTGGCTCTCGCTACCCGGGCCAAG
GCTCCCCGGGTGGCAACCGCTACCCACCGCAGGGCGGCGGCACGTGGGGTCAACCGCACGGTGGT
GGCTGGGGTCAACCGCACGGCGGTGGGTGGGGTCAACCTCACGGCGGCGGTTGGGGGCAACCGC
ACGGCGGTGGTTGGGGCCAGGGTGGTGGCACCCATAACCAGTGGAATAAGCCGAGCAAACCGAA
GACCAATATGAAGCATGTTGCAGGTGCTGCGGCGGCGGGTGCGGTGGTGGGTGGCCTGGGCGGTT
ATATGTTGGGCAGCGCAATGAGCCGTCCGATGATCCACTTTGGCAACGACTGGGAAGATCGTTAC
TACCGCGAGAACATGAATCGTTACCCGAATCAGGTGTATTATCGTCCGGTTGACCAGTATAACAAC
CAAAACAACTTCGTGCATGATTGTGTTAATATCACCATTAAACAACATACCGTAACTACGACGACC
AAAGGTGAGAACTTCACCGAAACCGACGTTAAAATGATGGAAAGAGTTGTCGAGCAGATGTGCGT
GACCCAGTACCAGAAAGAGAGCCAGGCCTATTACGAAGGTCGTTCGTAAAAGCTT -3’

<u>Amino acid sequence</u>
N-terminus-MKKRPKPGGWNTGGSRYPGQGSPGGNRYPPQGGGTWGQPHGGGWGQPHGG
GWGQPHGGGWGQPHGGGWGQGGGTHNQWNKPSKPKTNMKHVAGAAAAGAV
VGGLGGYMLGSAMSRPMIHFGNDWEDRYYRENMNRYPNQVYYRPVDQYNN
QNNFVHDCVNITIKQHTVTTTTKGENFTETDVKMMERVVEQMCVTQYQKE
SQAYYEGRS-**C terminus**

Table S1. Bank vole cDNA and amino acid sequence information.

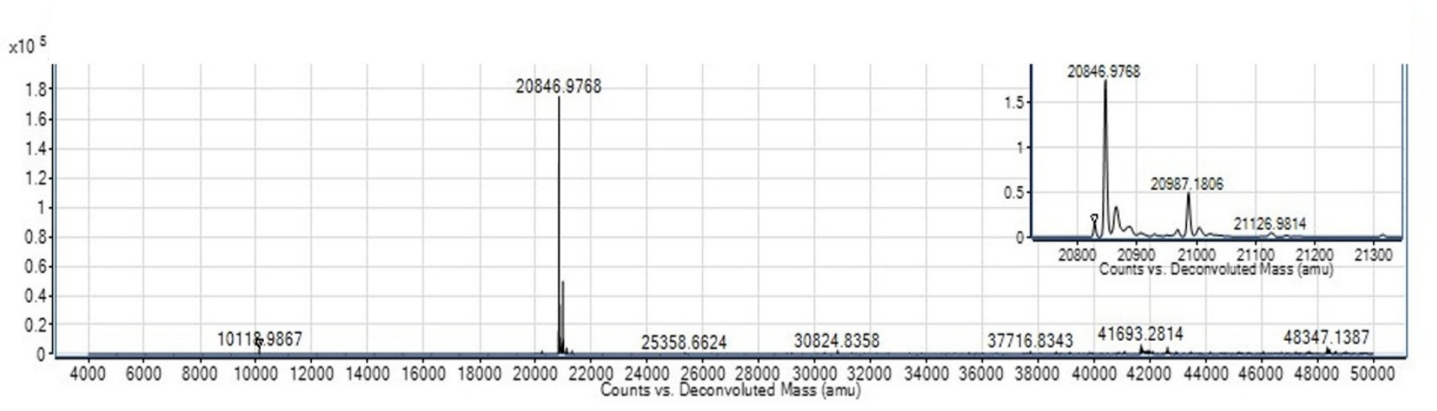

Figure S3. Electrospray ionization LC-MS deconvoluted mass spectrum of purified Bank vole prion protein in 25 mM Tris pH 8.0. Expected theoretical mass is 22,914.26 atomic mass unit (from ExPASy Peptide Mass tool (http://web.expasy.org/peptide_mass/) using average mass, with no cutting and $[M+H]^+$ options).

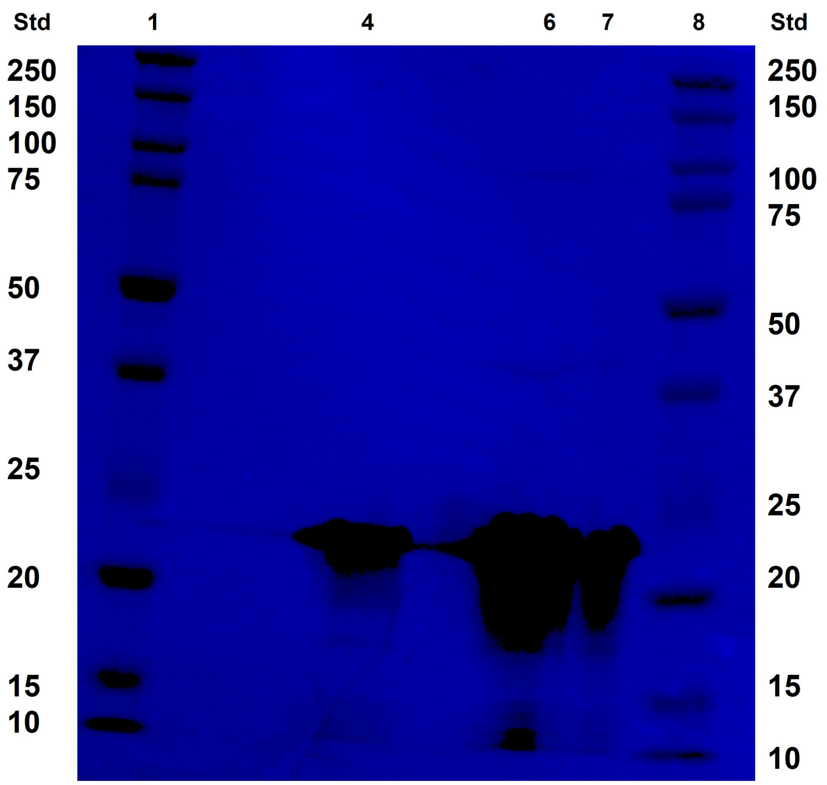

Figure S4. No heat SDS-PAGE of recombinant Bank vole prion protein (residues 23-231) stained with Coomassie blue. Lanes 1 and 8 are 10 µL of Biorad precision plus dual color standard, lane 4 is 100 kD filtered Bank vole rPrP at 15 µM used for RT-QuIC experiment, lanes 6 and 7 are unfiltered Bank vole rPrP at 40 µM and 15 µM.

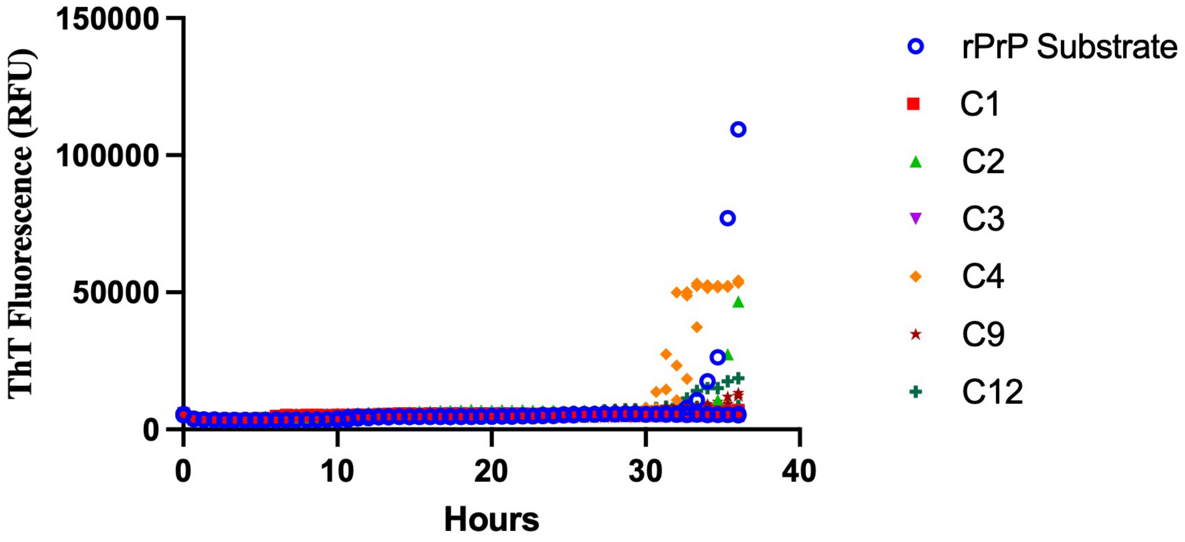

Figure S5. Prion RT-QuIC assay displaying lack of ThT signal in CWD negative samples and controls. CWD negative samples 1, 2, 3, 4 (deer lymph node), 9 (mouse brain *PRNP* knock-out), and 12 (WT deer brain) at 10^{-3} dilution.

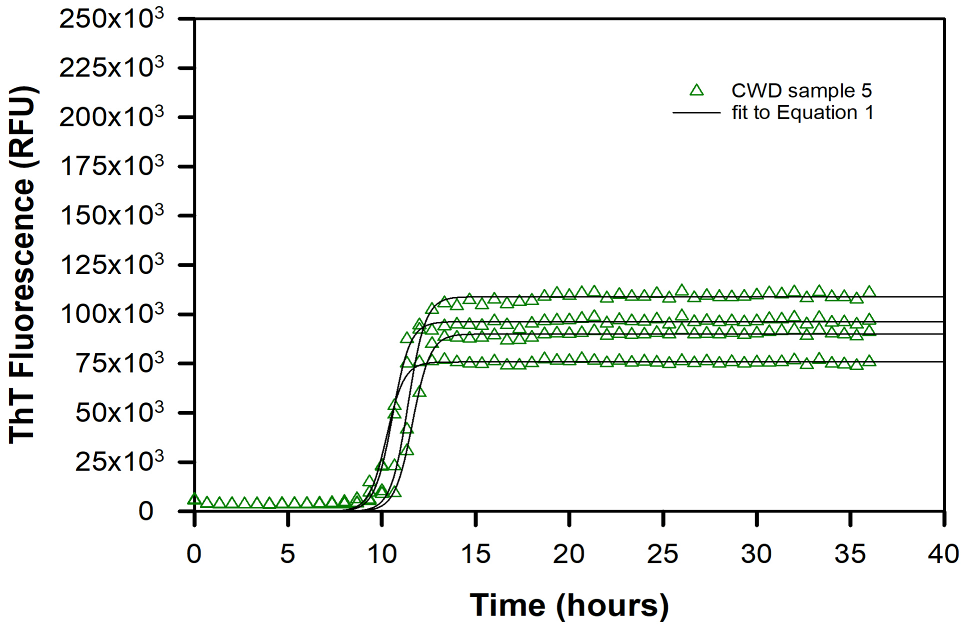

Figure S6. Representative figure of CWD positive data fitted to sigmoidal model (Eq 1 in main text) to estimate lag phase and ThT amplitude.

Polymorphic Codon	Reference Amino Acid	Variant Amino Acid	Reference Sequence	Variant Sequence	Variant Heterozygote Frequency (%)	Variant Homozygote Frequency (%)	CWD Positive (%)	Sample Numbers (out of 358)
25	Lysine (K)	Lysine (K)	AAG	AAA	100	0	0	1
108	Proline (P)	Proline (P)	CCA	CCG	0	100	0	1
131	Tyrosine (Y)	Tyrosine (Y)	TAC	TAT	93	7	26	96
139	Arginine (R)	Arginine (R)	AGG	AGA	0	100	0	4
156	Asparagine (N)	Asparagine (N)	AAT	AAC	0	100	25	6
185	Isoleucine (I)	Isoleucine (I)	ATC	ATT	0	100	0	1
202	Threonine (T)	Threonine (T)	ACT	ACC	17	83	25	6
237	Leucine (L)	Leucine (L)	CTC	CTG	100	0	0	1
238	Phenylalanine (F)	Phenylalanine (F)	TTC	TTT	83	17	18	12
247	Isoleucine (I)	Isoleucine (I)	ATC	ATT	93	7	31	14
253	Leucine (L)	Leucine (L)	CTC	CTT	100	0	0	1

Table S2: Synonymous *PRNP* polymorphisms identified in the dataset. Heterozygote, homozygote frequency and CWD percentages are within the polymorphic codon group.

Polymorphic Variant	Genbank ID (Accession number)
V2G R40Q	PZ139486
V12I A16P M17P Y52S	PZ123357
V12F	PZ123356
D20G R40Q	PZ139487
D20G T204N M209R S225Y A227G	PZ139637
G96S	PZ139635
T98I	PZ139636
T195S E210D C217R S225F F238L	PZ139489
M209R V213G C217S Y221S Q226R Y229S L237F	PZ139488

Table S3: Representative list of *PRNP* polymorphisms discovered within study, some of which carried multiple polymorphisms within the gene.

		AA p.V12I		AA p.V12F*		AA p.D20G		AA p.R40Q*		AA p.S225F		Wild-Type	
Year	Hunting Region	CWD +	CWD -	CWD +	CWD -	CWD +	CWD -	CWD +	CWD -	CWD +	CWD -	CWD +	CWD -
2017	401	0	0	0	0	0	2	0	0	0		0	21
	502	0	0	0	0	0	3	0	0	0	2	1	15
	555	0	0	1	0	0	6	0	1	0		1	9
	600	N/A		N/A		N/A		N/A		N/A		N/A	
	640	N/A		N/A		N/A		N/A		N/A		N/A	
	670	N/A		N/A		N/A		N/A		N/A		N/A	
2018	401	0	0	0	0	0	2	0	0	0	0	0	23
	502	N/A		N/A		N/A		N/A		N/A		N/A	
	555	N/A		N/A		N/A		N/A		N/A		N/A	
	600	0	0	0	0	2	3	0	0	0	0	3	13
	640	0	0	0	0	1	1	0	0	0	0	2	17
	670	0	0	0	0	0	2	0	0	0	0	3	15
2022	401	0	0	1	0	1	8	0	0	0	0	0	12
	502	0	0	0	0	0	4	0	1	0	0	3	10
	555	0	0	0	0	0	2	0	0	0	0	0	9
	600	0	0	0	0	1	0	0	1	0	0	6	7
	640	0	0	0	0	0	2	0	0	0	0	6	3
	670	1	0	0	0	1	2	0	0	0	0	8	8

Table S4: Mule deer non-synonymous *PRNP* polymorphisms (condensed dataset) integrated with CWD status in Montana hunting regions and year of harvest. *Novel mule deer *PRNP* polymorphisms are denoted with an asterisk. Red numbers indicate that at least one sample was suspected of CWD. Blue numbers indicate that at least one sample had multiple variants at another position which may affect total sample number. Samples found with synonymous polymorphisms not included. N/A indicates that no samples from those conditions are in the study. Samples labeled “suspect” were CWD positive on an initial ELISA assay, however, were not confirmed positive with a second test.

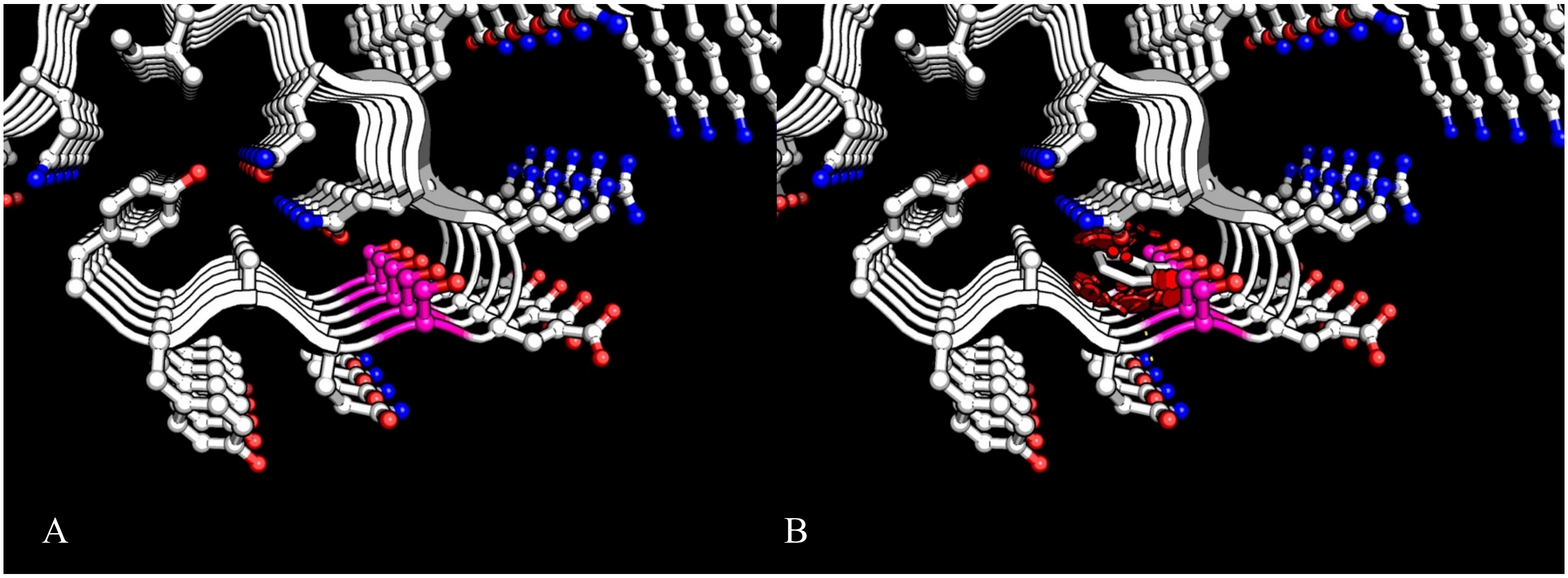

Figure S7: [A] 9DMY known fibril structure of wild type 225S genotype. [B] Steric clashes in the 9DMY fibril structure caused by the 225F genotype.

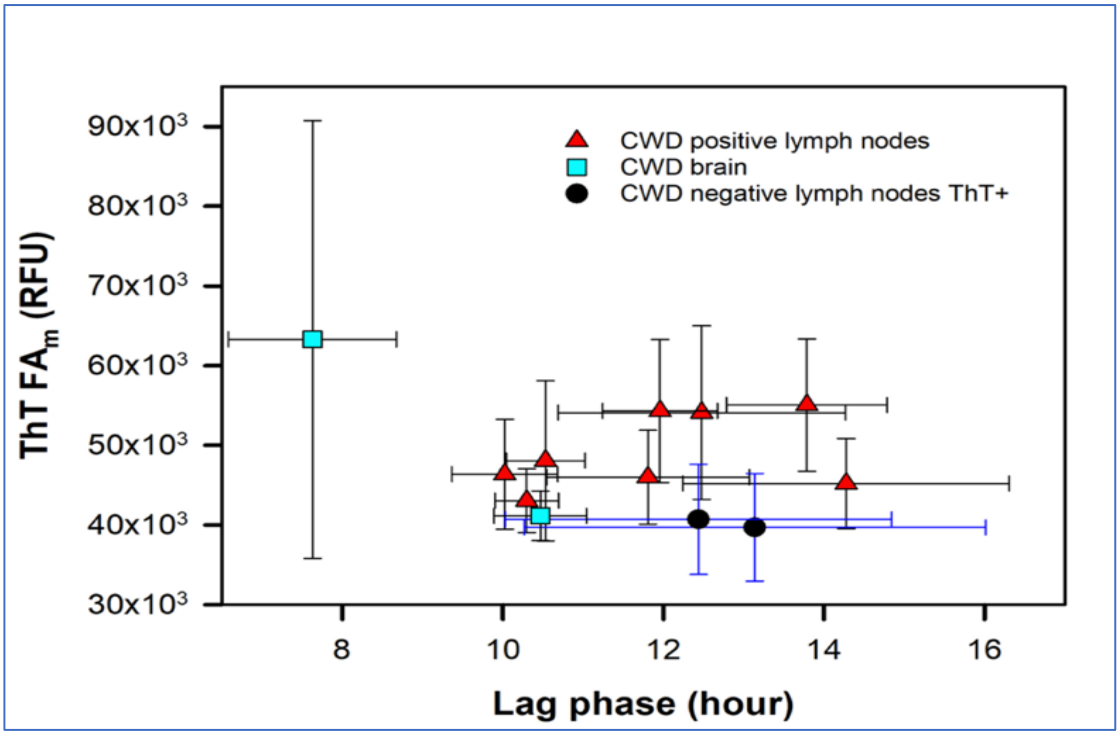

Figure S8: Lag phase (hours) versus half ThT fluorescence amplitude at the midpoint (ThT FA_m, RFU) for all ThT positive biospecimens via prion RT-QuIC.

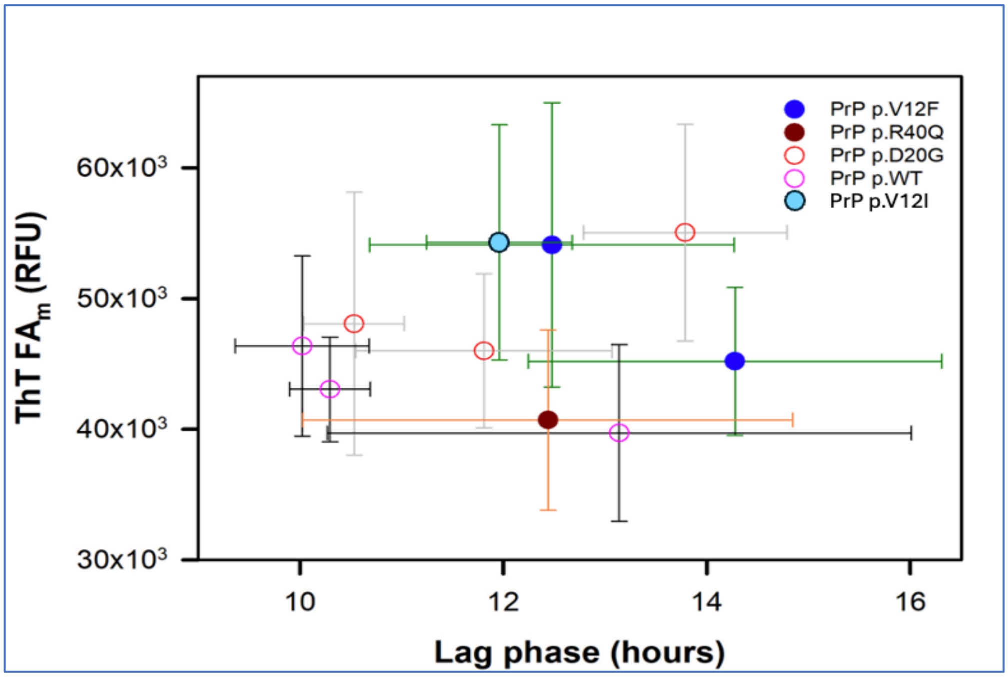

Figure S9. PrP variant and wild type sample comparisons of ThT fluorescence via lag phase (hours) and midpoint ThT fluorescence amplitude (ThT FA$_m$, RFU).

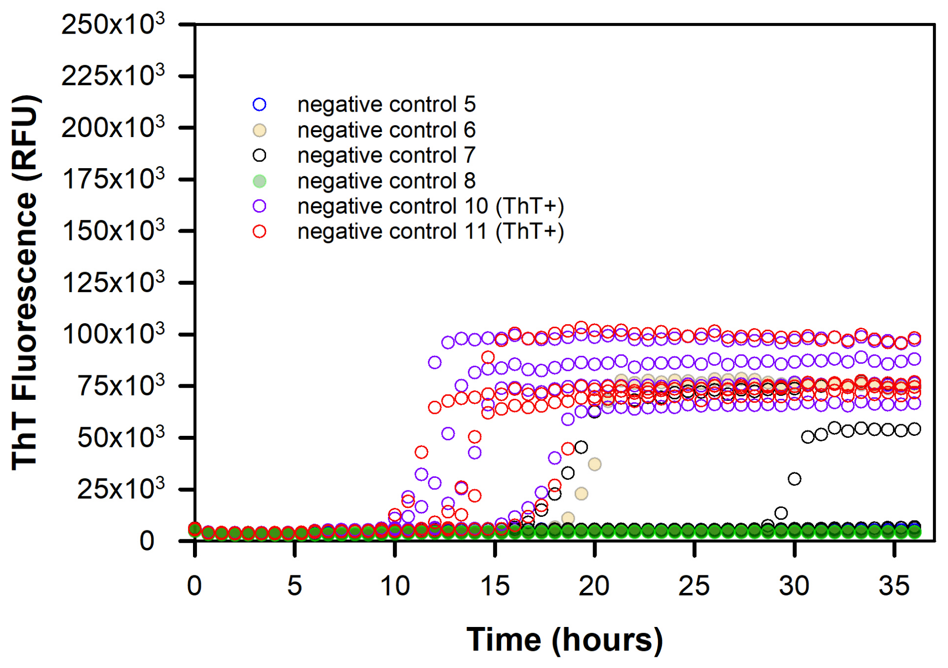

Figure S10. Prion RT-QuIC assay on samples 5, 6, 7, 8, 10, 11 (lymph node) all reported CWD negative by ELISA at 10^{-3} dilution. Replicates of samples 10 and 11 display ThT positive signal before 14.6 hours.
